# Distress criterion influences prevalence rates of functional gastrointestinal disorders

**DOI:** 10.1186/s12876-014-0215-9

**Published:** 2014-12-18

**Authors:** Charlotte Markert, Kerstin Suarez-Hitz, Ulrike Ehlert, Urs M Nater

**Affiliations:** Department of Psychology, University of Marburg, Gutenbergstrasse 18,35037, Marburg, Germany; Department of Psychology, University of Zurich, Binzmühlestrasse 14,8050, Zurich, Switzerland

**Keywords:** Distress, Functional gastrointestinal disorders, Prevalence rates, Rome criteria

## Abstract

**Background:**

Functional gastrointestinal disorders (FGID) are defined by a combination of chronic or recurrent gastrointestinal symptoms. Prevalence rates of FGID are high. Symptoms are associated with distress, and sufferers show high stress levels. However, the current diagnostic criteria do not consider subjective distress elicited by the symptoms, thus potentially leading to overestimated prevalence rates. The aim of this study was to explore the reduction in prevalence rates when distress is considered in the diagnostic criteria.

**Methods:**

In this web-based study, FGID were diagnosed using the Rome II criteria. Prevalence rates with and without subjective distress elicited by the symptoms were computed. Additionally, stress levels and stress reactivity were assessed.

**Results:**

Prevalence rates of FGID in our sample were similar to those in other studies. However, when considering the distress criterion, on average, a decrease of 38.51% was found in the prevalence rates of FGID. Sufferers who were subjectively distressed by their symptoms reported significantly higher stress levels than non-distressed subjects (all p < 0.001).

**Conclusions:**

The consideration of a criterion of subjective distress in the diagnosis of FGID has consequences for actual prevalence rates of FGID. Distressed subjects differ markedly from non-distressed subjects in terms of their stress levels. The inclusion of a distress criterion in the ongoing development of diagnostic criteria for FGID is therefore warranted.

**Electronic supplementary material:**

The online version of this article (doi:10.1186/s12876-014-0215-9) contains supplementary material, which is available to authorized users.

## Background

Functional gastrointestinal disorders (FGID) are defined by variable combinations of chronic or recurrent gastrointestinal symptoms that cannot be explained by structural or biochemical abnormalities [1]. In general, prevalence rates of FGID are high, ranging from 35% to 70% [1,2], with a considerable overlap between different syndromes [1]. A female preponderance has been found for most FGID [1,3,4]. Besides the exclusion of organic diseases by means of medical examination, the use of the Rome criteria is recommended to confirm an FGID diagnosis. Updated versions of the original Rome criteria of 1994 [5] were published in 1999 (Rome II [6]) and in 2006 (Rome III [7]). The prevalence rates of FGID depend on various sample characteristics such as sex, ethnicity, socio-economic status, and lifestyle factors [1,2]. Moreover, prevalence rates vary depending on the diagnostic criteria used, with the current Rome III criteria being less restrictive than the Rome II criteria with respect to symptom duration [8,9]: Whereas the Rome II criteria include a symptom duration of 12 months, the Rome III criteria take into account a 6-month duration. There is stronger evidence for the validity of the Rome II criteria [10], which are more frequently used in clinical research [11].

To date, the pathophysiology of FGID remains elusive, and treatment options are limited. Current knowledge suggests that FGID etiologies are biopsychosocial in nature. Given that psychological factors play a significant role in FGID, it is interesting to note that these are not considered in the diagnosis of FGID. In essence, diagnosis is reached by summarizing specific symptoms that have been present for a certain amount of time and for which there is no medical explanation. Importantly, however, FGID cause distress in patients struck by the illness, with some patients suffering more than others.

An important question therefore refers to how distress is defined. A great number of studies quantified the level of subjective distress using measures of quality of life. However, in all of these studies, distress was defined as psychological distress related to anxiety and depression. The lack of a uniform application of a distress measure in FGID is particularly striking for researchers and practitioners in the field of psychiatry, as it is a well-established standard in psychiatric diagnostic criteria using the Diagnostic and Statistical Manual of Mental Disorders (DSM-IV). For the diagnosis of psychiatric disorders according to the DSM-IV, clinical significance is only given if a patient reports being subjectively distressed by his or her symptoms. As yet, the impact of subjective distress elicited by the symptoms on the prevalence rates of FGID has not been investigated, even though such a consideration might have clinically relevant consequences.

Diagnoses made according to the Rome II criteria or the current Rome III criteria do not consider subjective distress elicited by the symptoms. It seems important, however, to only diagnose those individuals with FGID who are actually suffering from their symptoms. In studies investigating one particular FGID, i.e. irritable bowel syndrome (IBS), no statistically significant differences were found between persons who were seeking medical advice and persons who were not seeking medical advice with regard to the number or severity of complaints, despite the fact that all individuals fulfilled the full criteria for IBS [12,13]. This implies that some persons do not seem to be sufficiently bothered by the number and severity of their gastrointestinal symptoms to seek medical advice. It may therefore be questioned whether their “illness” is actually clinically relevant if they do not feel the need to do something about it. Despite this, persons who do not find their symptoms bothersome are also included in epidemiological studies of FGID prevalence rates. Accordingly, the findings from population-based studies cannot be generalized to persons seeking medical care.

As noted above, psychological factors have been identified as playing an important role in FGID, with stress being assumed to play a central role in its development [14].

We describe subjective stress in accordance with Lazarus et al., who stated that stress is not defined by the objective requirements of the situation, but by the person’s subjective evaluation of the situation and his/her strategies to cope with it [15]. If a person perceives situations as stressful over a longer period of time, subjective stress may result in chronic stress. According to the allostatic load model [16], chronic stress may ultimately lead to wear and tear on the body, with the consequence of stress-related conditions such as FGID or certain psychiatric disorders. With regard to FGID, studies found that the amount of events a person perceives as stressful is linked to the presence of gastrointestinal symptoms [17,18]. Moreover, subjects with FGID report more negative life events as well as fewer positive life events [18-20]. These findings highlight the importance of perceived stress in FGID.

Chronic stress is often mentioned together with anxiety and depression traits, but the terms should not be used interchangeably. Anxiety and depression can be defined as psychopathological phenomena, which show common characteristics with chronic stress (e.g. negative affectivity, dysregulation of stress hormones).

Moreover, in laboratory studies, it was found that persons with FGID show a greater psychological and biological reactivity to stressors, which might influence the perception of gastrointestinal discomfort and pain [21-23]. A previous online study, which examined both chronic stress and stress reactivity, found that even young subjects with FGID report higher levels of chronic stress and stress reactivity compared to controls [24]. In sum, these studies show that people with FGID have different stress experiences and react differently to stressors compared to healthy controls.

Therefore, examining potential differences in stress levels between individuals who are distressed by their symptoms and those who are not would appear to be of importance. It is hypothesized that being distressed by one’s symptoms may play a critical role in whether or not one receives a clinically meaningful diagnosis of FGID. The aim of our study was twofold: 1) We investigated potential differences in prevalence rates between people fulfilling the Rome II criteria for FGID and people fulfilling both the Rome II criteria and a subjective distress criterion, and 2) we examined differences in chronic stress and stress reactivity between these groups and compared both to a healthy control group. We decided to test our hypothesis in a relatively homogenous group of young individuals. Should we find that the inclusion of a distress criterion has an impact on FGID prevalence rates in this population, the necessity of this approach could be tested in a clinical sample of patients.

## Methods

### Participants and procedure

Data were collected in 2004 in the German-speaking part of Switzerland. All potential participants from two universities (University of Zurich and Federal Institute of Technology Zurich) were contacted via e-mail and asked to take part in a survey on stress and bodily symptoms. Participation was voluntary and all subjects provided electronic informed consent. Anonymity was guaranteed by automatically sending a username, a password and an internet link to the survey to the participants. Further psychometric data and subjects’ characteristics were saved separately. In the case of acute psychological problems, clinic contact information was provided. All procedures were in accordance with the ethical standards laid down in the 1964 Declaration of Helsinki, and the web survey study design was approved by the ethics committee of the Canton of Zurich. Written informed consent was obtained from all participants.

The complete online survey consisted of 178 items. Participants had to answer all of the questions on each page of the survey in order to continue to the next page. This enabled complete datasets to be collected. As compensation for their effort, participants had a chance to win cinema tickets and a dining coupon. The study was carried out under conditions in accordance with the Declaration of Helsinki.

### Measurements

In the first part of the online survey, sociodemographic and health-related data (e.g. diseases, medication, consultation rates, use of addictive drugs) were collected. A dichotomous question was used to assess whether participants regularly visited the doctor (“regular doctor visits”: “yes/no”). If organic diseases were reported which were potentially related to gastrointestinal symptoms, participants were excluded from the calculation of prevalence rates.

Subsequently, the following questionnaires about FGID (including an assessment of subjective distress) and stress were applied:

- The *Gastro-Questionnaire* [25], which allows the assessment of 27 gastrointestinal symptoms, was administered to analyze the prevalence of twenty FGID (Additional file 1: Table S1) according to the Rome II criteria [6]. The occurrence of each symptom was rated on a 4-point scale: 0 (not at all), 1 (from time to time), 2 (frequently), and 3 (nearly always). The level of subjective distress for each symptom was rated on a 5-point scale from 0 (no distress) to 4 (very severe distress). Persons who fulfilled the Rome II criteria for an FGID and graded the distress for one symptom of this disorder with values of 2 or higher were classified as “distressed subjects”.
- The *Trier Inventory for the Assessment of Chronic Stress (TICS)* [26] was included to gather information about subjectively perceived stress within the last 12 months. This self-report questionnaire consists of 57 items concerning eight types of chronic stress (work overload, social work overload, overextended at work, lack of social recognition, work discontent, social tension, pressure to succeed, social isolation).
- The *Stress Reactivity Scale (SRS)* [27,28] is an instrument which measures reactivity to potentially stressful situations. A three-level response format is specified for each of the 36 items. In addition to general stress reactivity, the scale also assesses stress reactivity in specific domains (Reactivity to Work Overload, Reactivity to Social Conflicts, Reactivity to Social Stress, Reactivity to Failure, Anticipatory Reactivity, Prolonged Reactivity).

### Statistical analysis

Prevalence rates of FGID with and without distress according to the Rome II criteria were calculated in percentages. Before univariate analyses of variance were computed, data were tested for normal distribution using the Kolmogorov-Smirnov test and by calculating skewness and kurtosis of the distribution. A significant deviation from normal distribution is assumed if values for skewness are | ≤ 2| and values for kurtosis are | ≥ 7| [29]. According to the definition by West et al. [29], normal distribution was assumed for these data. Additionally, data were tested for homogeneity of variance using the Levene’s test. Homogeneity of variance was violated in some cases, leading to more conservative results, with the exception of the scale “stress reactivity before stressors”, which showed a more liberal result. Subsequently, univariate analyses of variance were calculated for the comparison of scale means. For statistical analysis, SPSS version 20 was used.

## Results

### Sample characteristics

In total, 1901 participants completed the web survey. Forty-four (2.31%) participants had to be excluded from the calculations because they reported an organic disease which might have explained their gastrointestinal symptoms. Thus, a total of 1857 participants were included in the analysis. One thousand and sixty-eight (57.50%) were women and 789 (42.50%) were men, and the mean age was 23.9 years (SD = 3.96). As all participants were university students, they were comparable with regard to their level of education.

### Prevalence rates of FGID

Additional file 1: Table S1 shows prevalence rates of FGID diagnoses according to the Rome II criteria alone compared to prevalence rates of FGID diagnoses with additional consideration of being distressed by the symptoms. Prevalence rates of FGID diagnoses according to the Rome II criteria without consideration of subjective distress were also reported in Suarez, Herdener-Pinnekamp, Ehlert, and Nater (under review). 1166 (62.79%) fulfilled the Rome II criteria for at least one diagnosis. The number of diagnoses ranged from one to six, with 548 (29.51%) persons reporting only one FGID, 379 (20.41%) reporting two, 157 (8.45%) reporting three, 62 (3.34%) reporting four, 18 (0.97%) reporting five, and two persons (0.11%) reporting six FGID diagnoses occurring concomitantly. When the distress criterion was taken into account, the prevalence rates changed as follows: 717 participants (38.61%) fulfilled the Rome criteria for at least one diagnosis. The number of diagnoses ranged from one to six FGID, with 399 (21.5%) persons reporting only one FGID, 196 (10.55%) reporting two, 78 (4.20%) reporting three, 34 (1.83%) reporting four, 9 (0.48%) reporting five and one person (0.05%) reporting six FGID diagnoses occurring concomitantly.

Thus, on average, a 38.51% decrease in prevalence rates was observed across all FGID diagnoses and all participants when subjective distress elicited by the symptoms was additionally considered. The largest differences were found for functional diarrhea (100% lower prevalence rate), aerophagia (77.08%), chronic functional abdominal pain (68.99%), and functional abdominal bloating (61.13%). No changes or only small reductions in the prevalence rates were found for proctalgia fugax (0%), functional incontinence (1.52%), and functional constipation (2.5%). Those changes were more distinct in men than in women (lower prevalence rates in 13 of the 21 syndromes in men compared to lower prevalence rates in 6 of the 21 syndromes in women and identical prevalence rates in 2 of the 21 syndromes).

There were 691 participants who did not fulfill any FGID diagnosis (healthy control group, HCG). In the next step, participants who fulfilled at least one diagnosis according to the Rome II criteria (n = 1166) were divided into a distressed (n = 717) and a non-distressed (n = 449) subgroup (see above). A group comparison indicated that there was a higher proportion of women in the distressed subgroup (66.10%) than in the non-distressed subgroup (56.1%) and the HCG (49.50%) (χ^2^ (2, n = 1068) = 70.05, p < .001). No significant differences were observed with regard to age (distressed subgroup: mean age = 23.88 years; non-distressed subgroup: mean age = 23.89 years; HCG: mean age = 23.86 years; F = .007; p = 0.993).

The three groups differed regarding clinical variables. The distressed subgroup reported significantly more symptoms than the non-distressed subgroup and the HCG (distressed subgroup: mean value of symptoms = 4.77; non-distressed subgroup: mean value of symptoms = 2.86; HCG: mean value of symptoms = 0.51; F = 557.86; p < 0.001). In the distressed subgroup, there were more persons undertaking regular doctor visits (23.80%) as compared to the non-distressed subgroup (18.90%) and the HCG (13.50%) (χ^2^ (2, n = 349) = 38.81, p < .001). Less than one third (29.93%) of all participants with at least one FGID diagnosis visited the doctor regularly.

Significant differences between the three subgroups were also observed with respect to chronic stress and stress reactivity (Additional file 2: Table S2) for all subscales of the TICS and the SRS, with the highest values in all subscales for the distressed group, followed by the non-distressed group and the HCG. Analyses separated by gender led to comparable results for men and women, with the exception that men showed slightly different means in two TICS scales (Social Tension: F = 4.36, p < 0.05; SRS, Reactivity to Social Conflicts: F = 3.91, p < 0.05).

## Discussion

The purpose of this study was to determine changes in prevalence rates of FGID in a non-clinical sample when subjective distress elicited by the symptoms is taken into account. Moreover, it was of interest to ascertain whether the subjects with FGID diagnoses and subjective distress exhibit higher levels of chronic stress and stress reactivity than those without subjective distress.

The results indicate that prevalence rates of FGID according to the Rome II criteria were high in our sample, with 62.78% of the subjects fulfilling at least one FGID diagnosis. This is comparable to population-based studies [1,30,31]. Studies which examined student samples found comparable prevalence rates [24] or slightly lower rates (51.2%) of FGID diagnoses [30]. Differences in prevalence rates may be due to the use of different assessment instruments for diagnosing FGID.

When a subjective distress criterion was considered, the prevalence rates of FGID deviated considerably from the original prevalence rates, with 38.6% of the sample fulfilling the criteria for at least one diagnosis. On average, this constitutes a decrease of 38.51% across all FGID and all participants. This decrease varies widely depending on the individual syndrome. In particular, the criteria for aerophagia, chronic functional abdominal pain and functional abdominal bloating were easily fulfilled, but less than half of the affected participants reported subjective distress elicited by the symptoms. Other FGID showed only a slight decline in prevalence rates, which may be attributable to the specific symptoms themselves (e.g. functional chest pain could be interpreted as more threatening than other symptoms) or to a possibly differing psychosocial relevance (e.g. the shame caused by functional incontinence). Men showed more severe drops in prevalence rates in 13 of the 21 syndromes. As the case numbers for some FGID in this study were only small, these findings need to be replicated in large population studies.

Due to the great impact that the consideration of subjective distress exerts on FGID prevalence rates, it might be important to incorporate a subjective distress criterion into the future Rome criteria, similar to the psychiatric diagnostic criteria in the DSM-IV. Failing to consider the distress criterion might potentially lead to an overestimation of prevalence rates of FGID, because diagnoses are only made based on positive symptom lists and exclusion of physical illness, while neglecting to acknowledge the degree of distress caused by the symptoms. It might be speculated that the above-mentioned high prevalence rates of FGID in population-based and student samples [1,24,30,31] indicate that the current FGID diagnostic criteria are over-inclusive. A recently published study recommended the consideration of multi-axial criteria in the diagnosis of FGID [33]. Distress elicited by the symptoms could be one of these criteria for measuring the impact of the symptoms on the patient’s life.

In this non-clinical study, healthy subjects showed lower values of chronic stress compared to subjects with at least one FGID diagnosis, indicating a relationship between self-reported gastrointestinal symptoms and work-related or social stress. These findings are comparable to our previous results in another, independent sample of students (Suarez et al., [24]). Importantly, however, subjects with at least one FGID diagnosis who also reported that they were subjectively distressed by their symptoms showed the highest values in each subscale of the *Trier Inventory for the Assessment of Chronic Stress,* indicating high stress levels during the past year. The positive association between FGID (especially IBS) and chronic stress is well known. Earlier studies found that groups with IBS showed greater levels of daily stress compared to controls [17,34,35]. Our study suggests that there is a relationship between the level of chronic stress and the subjective distress caused by the symptoms. Although the causal direction of this relationship remains unclear, it is conceivable that increased chronic stress could exacerbate or facilitate the maintenance of gastrointestinal symptoms which are subjectively distressing. It is also possible, however, that perceived distress elicited by the symptoms may ultimately lead to chronic stress in other areas of life. Studies with a longitudinal design are needed to examine this causal relationship.

A similar pattern can be found regarding stress reactivity. Subjects with at least one self-reported FGID diagnosis showed increased scores in each subscale of the *Stress Reactivity Scale*. Again, this finding is in accordance with the results of our previous study (Suarez et al., [24]), and the results are in line with previous studies indicating increased reactivity to stress in patients with IBS [21,22]. In our current study, we found that stress reactivity was highest in those individuals with FGID who also felt distressed by their symptoms, followed by non-distressed subjects with FGID and healthy controls. While our data indicate that there is a relationship between the level of stress reactivity and subjective distress, due to the correlational design of the study, the causal direction of this relationship remains unclear. It is thus uncertain whether increased stress reactivity leads to more distress due to the symptoms or whether persons who are more distressed by their symptoms show a higher reactivity to stress in other areas of life. Longitudinal designs are needed to answer this question.

This study aimed to assess the impact of including a subjective distress criterion in the diagnosis of FGID on the prevalence rates of FGID. To the best of our knowledge, this aspect has not been previously examined. Despite various advantages, some limitations need to be taken into account when interpreting the study results: The voluntary nature of participation may have led to a self-selection bias, and as the subjects examined in the sample did not undergo specific physical investigations, a validation of the self-reported diagnoses is lacking. Furthermore, the study should be repeated using the current Rome III criteria in order to determine whether our findings apply to these less conservative criteria as well. However, the decrease in prevalence rates is likely to be even larger in studies using the current Rome III criteria due to the shorter symptom duration, meaning that our results may be too conservative. We collected data from a student sample, which cannot be considered as representative of the general Swiss population regarding e.g. age, level of education, socioeconomic status, and lifestyle factors. Most population-based studies on FGID included patients aged between 40 and 50 [12,36,37]. However, various studies have demonstrated that FGID are also prevalent in younger individuals [24,38-40]. Thus, while our findings are not representative of the majority of potential FGID cases, our sample may provide important insights into younger FGID patients.

We assume that biological, psychological, and social aspects play a role in the development and maintenance of FGID. Stress can cause a reduction in pain thresholds, resulting in abdominal pain [41]. It can be assumed that biomarkers related to stress, e.g. corticotropin-releasing hormone (CRH), influence intestinal health and affect intestinal motility in patients with functional gastrointestinal syndromes [42]. Several studies [43-45] showed that microbiota in IBS differ from microbiota in healthy controls, and may therefore play an important role in the etiology of IBS and other FGID. Future research is needed to gain an understanding of the complex brain-gut interactions, involving both endocrine pathways and intestinal microbiota. With regard to our research focus, it would be of great interest to ascertain whether subjects who fulfill the distress criterion have different biological alterations than those who do not.

Moreover, since cultural attitudes influence the openness to report symptoms [46,47], these findings need to be replicated in other cultural contexts.

Finally, we did not examine subjective beliefs, such as catastrophizing or fear avoidance, in this study. Since these psychological aspects are included in the new DSM-5 criteria, they are becoming increasingly important. However, the psychological criteria are not independent of each other and there is a lack of precise guidelines on how to assess them [48]. For one specific FGID, i.e. irritable bowel syndrome, the effect of catastrophizing on pain severity and pain-related suffering is well documented [49-51]. Future studies should examine whether these psychological aspects are related to the distress elicited by one’s symptoms.

For the practitioner, we recommend simply asking patients which symptoms cause distress by using a 5-point Likert scale from 0 (no distress) to 4 (very severe distress) in the same way as it is done in the “Gastro-Questionnaire”, which was used in the current study and of which an English version exists [25]. Persons who fulfill the current Rome Criteria for an FGID and who grade the distress for at least one symptom as 2 or higher (moderate distress) may then be classified as having an FGID diagnosis with distress. In addition, it would be desirable to acquire information about chronic stress, e.g. using the Screening Scale for Chronic Stress (SSCS) [26] which consists of only 12 items.

## Conclusion

The study revealed valuable information about the impact of subjective distress on the prevalence rates of FGID. We therefore suggest that a criterion of subjective distress should be taken into consideration during the Rome IV process. Moreover, our results might be important for clinical practice because individuals who are distressed by their symptoms are more likely to also present increased stress levels. Stress management training could be particularly beneficial for patients who report distress due to their symptoms.

## Additional files

Additional file 1:
**FGID diagnoses with and without subjective distress for the complete sample and for each gender.**
Additional file 2:
**Differences in chronic stress and stress reactivity between the three subgroups.**

## Abbreviations

FGID: Functional gastrointestinal disorders
IBS: Irritable bowel syndrome
TICS: Trier Inventory for the Assessment of Chronic Stress
SRS: Stress Reactivity Scale
SD: Standard deviation
HCG: Healthy control group
DSM-IV: Diagnostic and Statistic Manual of Mental Disorders IV
