# Supplementary material for: Distress criterion influences prevalence rates of functional gastrointestinal disorders
Source: BMC Gastroenterol. 2014 Dec 18;14:215. doi: 10.1186/s12876-014-0215-9 (PMC4284923; doi:10.1186/s12876-014-0215-9)
Supplement: Additional file 1: — FGID diagnoses with and without subjective distress for the complete sample and for each gender. [file 12876_2014_215_MOESM1_ESM.pdf]

Table 1: FGID diagnoses with and without subjective distress for the complete sample and for each gender

Diagnoses according to Rome II												
**Diagnosis**	**Absolute cases**	**%**	**Absolute cases (distressed)**	**%**	**Women**	**%**	**Women (distressed)**	**%**	**Men**	**%**	**Men (distressed)**	**%**
**Globus syndrome**	58	3.12	36	1.94	40	3.75	26	2.43	18	2.28	10	1.27
**Functional chest pain**	48	2.58	42	2.26	31	2.90	28	2.62	17	2.15	14	1.77
**Functional heart burn**	25	1.35	11	0.59	10	0.94	3	0.28	15	1.90	8	1.01
**Functional dysphagia**	20	1.08	16	0.86	14	1.31	12	1.12	6	0.76	4	0.51
**Unspecified functional esophageal disorder**	160	8.62	122	6.57	107	10.02	82	7.68	53	6.72	40	5.07
**Functional dyspepsia (ulcer-like)**	151	8.13	142	7.65	120	11.24	112	10.49	31	3.93	30	3.80
**Functional dyspepsia (dysmotility-like)**	80	4.31	58	3.12	63	5.90	49	4.59	17	2.15	9	1.14
**Functional dyspepsia**	45	2.42	42	2.26	32	3.00	31	2.90	13	1.65	11	1.39

**(reflux-like)**												
**Functional dyspepsia (unspecified)**	116	6.25	59	3.18	87	8.15	44	4.12	29	3.68	15	1.90
**Aerophagia**	144	7.75	33	1.78	65	6.09	16	1.49	79	10.01	17	2.15
**Irritable bowel syndrome**	84	4.52	48	2.58	64	5.99	34	3.18	20	2.53	14	1.77
**Irritable bowel syndrome (diarrhea predominant)**	66	3.55	39	2.10	49	4.59	27	2.53	17	2.15	12	1.52
**Irritable bowel syndrome (constipation predominant)**	40	2.15	22	1.18	38	3.56	21	1.97	2	0.25	1	0.13
**Functional constipation**	40	2.15	39	2.10	35	3.28	35	3.28	5	0.63	4	0.51
**Functional diarrhea**	4	0.22	0	0.00	1	0.09	0	0.00	3	0.38	0	0.00
**Functional abdominal bloating**	705	37.96	274	14.75	409	38.30	172	16.10	296	37.52	102	12.93
**Unspecified functional bowel disorder**	175	9.42	137	7.38	139	13.01	109	10.21	36	4.56	28	3.55

**Chronic functional abdominal pain**	158	8.51	49	2.64	108	10.11	36	3.37	50	6.34	13	1.65
**Functional incontinence**	66	3.55	65	3.50	30	2.81	30	2.81	36	4.56	35	4.44
**Levator syndrome**	44	2.37	35	1.88	19	1.78	14	1.31	25	3.17	21	2.66
**Proctalgia fugax**	4	0.22	4	0.22	3	0.28	3	0.28	1	0.13	1	0.13

The prevalence rates of the two subgroups of irritable bowel syndrome do not result in the total prevalence of irritable bowel syndrome, because some participants could not be allocated to a subgroup or they met the criteria of both subgroups concomitantly.
