# Supplementary material for: Distress criterion influences prevalence rates of functional gastrointestinal disorders
Source: BMC Gastroenterol. 2014 Dec 18;14:215. doi: 10.1186/s12876-014-0215-9 (PMC4284923; doi:10.1186/s12876-014-0215-9)
Supplement: Additional file 2: — Differences in chronic stress and stress reactivity between the three subgroups. [file 12876_2014_215_MOESM2_ESM.pdf]

Table 2: Differences in chronic stress and stress reactivity between the three subgroups

	Descriptive statistics			Analysis of variance	
Scale	Healthy control group Mean (SD) n=691	Non-Distressed group Mean (SD) n=449	Distressed group Mean (SD) n=717	F	Significance (p)
**TICS: Work overload**	13.81 (5.65)	14.28 (5.94)	16.38 +¤ (6.01)	37.35	< 0.001
**TICS: Social work overload**	6.63 (3.87)	6.63 (4.03)	7.85 +¤ (4.30)	19.90	< 0.001
**TICS: Pressure to succeed**	15.02 (4.92)	15.43 (5.17)	16.94 +¤ (5.30)	26.53	< 0.001
**TICS: Work discontent**	10.68 (5.16)	11.43 (4.94)	12.24 +◊ (5.32)	16.08	< 0.001
**TICS: Overextended at work**	6.39 (3.69)	6.74 (3.96)	8.20 +¤ (4.07)	41.68	< 0.001
**TICS: Lack of social recognition**	4.04 (2.59)	4.36 (2.73)	5.31 +¤ (3.08)	38.06	< 0.001
**TICS: Social tension**	5.16 (3.65)	5.49 (3.31)	6.49 +¤ (3.77)	25.28	< 0.001
**TICS: Social isolation**	7.24 (4.87)	7.64 (4.86)	8.55 +◊ (5.35)	12.16	< 0.001
**TICS: Worries**	6.10 (3.40)	6.72 † (3.40)	8.20 +¤ (3.59)	67.28	< 0.001
**TICS: screening scale for chronic stress**	15.59 (7.31)	16.88 † (7.39)	20.59 +¤ (7.70)	83.43	< 0.001
**SRS: Reactivity to work overload**	8.36 (2.309	9.06 ¥ (2.40)	9.69 +¤ (2.32)	56.88	< 0.001

**SRS: Reactivity to social conflicts**	12.63 (2.55)	12.98 (2.40)	13.59 [+¤] (2.51)	26.49	< 0.001
**SRS: Reactivity to social stress**	9.40 (2.33)	9.93 [Δ] (2.37)	10.57 [+¤] (2.34)	44.26	< 0.001
**SRS: Reactivity to failure**	10.42 (1.90)	10.60 (1.90)	11.17 [+¤] (1.96)	28.35	< 0.001
**SRS: Anticipatory reactivity**	8.35 (1.84)	8.67 [†] (1.79)	9.14 [+¤] (1.67)	36.07	< 0.001
**SRS: Prolonged reactivity**	6.15 (1.82)	6.39 (1.79)	7.09 [+¤] (2.09)	43.94	< 0.001
**SRS: Overall stress reactivity score**	55.31 (9.04)	57.64 [¥] (8.83)	61.24 [+¤] (8.94)	78.26	< 0.001

[+] significant difference between participants with at least one Rome II diagnosis with consideration of subjective distress and healthy participants (α < 0.001)
[¤] significant difference between participants with at least one Rome II diagnosis with consideration of subjective distress and participants with at least one Rome II diagnosis without distress (α < 0.001)
[◊] significant difference between participants with at least one Rome II diagnosis with consideration of subjective distress and participants with at least one Rome II diagnosis without distress (α < 0.05)
[¥] significant difference between participants with at least one Rome II diagnosis without distress and healthy participants (α < 0.001)
[Δ] significant difference between participants with at least one Rome II diagnosis without distress and healthy participants (α< 0.01)
[†] significant difference between participants with at least one Rome II diagnosis without distress and healthy participants (α < 0.05)
